# Quantification of Acipimox in Plasma and Tissues by LC–MS/MS: Application to Pharmacokinetic Comparison between Normoxia and Hypoxia

**DOI:** 10.3390/molecules27196413

**Published:** 2022-09-28

**Authors:** Xin Shen, Gaofu Li, Libin Wang, Huijin Yu, Lei Zhou, Huifang Deng, Ningning Wang, Chengcai Lai, Wei Zhou, Yue Gao

**Affiliations:** 1 Department of Pharmaceutical Sciences, Beijing Institute of Radiation Medicine, Beijing 100850, China; 2 School of Medicine, Shaanxi Energy Institute, Xianyang 712000, China; 3 School of Pharmacy, Guangdong Pharmaceutical University, Guangzhou 510006, China

**Keywords:** LC–MS/MS, hypoxia, acipimox, pharmacokinetics

## Abstract

This study aimed to evaluate the pharmacokinetics of acipimox in rats under simulated high altitude hypoxia conditions. A sensitive and reliable LC–MS/MS method has been established for the quantitation of acipimox in rat plasma and tissue homogenate and validated according to the guidelines of the European Medicines Agency (EMA) and the US Food and Drug Administration (FDA). Western blotting and enzyme linked immunosorbent assay (ELISA) were used to investigate the expression of lipid metabolism-related proteins and free fatty acid (FFA) levels, respectively. Cell viability was detected using a Cell Counting kit-8 assay (CCK-8). The method was then successfully applied in a pharmacokinetic comparison between normoxic and hypoxic rats. The results indicated that there were significant differences in the main pharmacokinetics parameters of acipimox between normoxic and hypoxic rats. HCAR2 expression in the hypoxia group was upregulated compared to that in the normoxia group and the levels of FFA decreased more in the hypoxia group. Under the hypoxia condition, the proliferation of HK2 cells was inhibited with increasing concentrations of acipimox. The results provide important and valuable information for the safety and efficacy of acipimox, which indicated that the dosage of acipimox might be adjusted appropriately during clinical medication in hypoxia.

## 1. Introduction

The environmental characteristics of high altitude areas are hypoxia, low pressure, low temperature, dry air, and strong ultraviolet radiation, in which hypoxia plays a major role in the changes of physiological functions [1]. Under a plateau environment, inadequate oxygen makes people breathe less oxygen, reducing the level of oxygen metabolism and energy supply in the body [2,3]. Subsequently, the peripheral circulation, the contractile efficiency of myocardial cells, the pump of the bloodstream, the flow rate of blood in various tissues, and the excretion rate of waste in the body could be greatly reduced, which causes metabolic disorders and mountain sickness [4,5,6]. Studies have shown that hypoxia could also affect the activities of drug metabolic enzymes, transporters, renal blood flow and intestinal flora, etc., which might affect the metabolism and efficacy of drugs [7,8,9,10].

**Figure 1:**
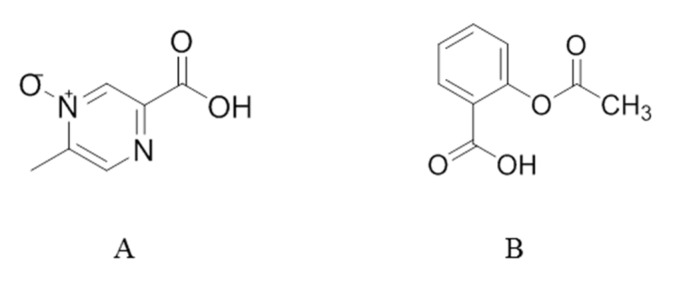
Chemical structures of the analytes: acipimox (**A**) and the internal standard (IS) acetylsalicylic acid (**B**).

Acipimox (Figure 1), a derivative of nicotinic acid, has been used for almost 50 years as a lipid-lowering drug. The target of acipimox is hydroxycarboxylic acid receptor 2 (HCAR2), which can inhibit fat mobilization and lipid synthesis by reducing intracellular cAMP concentrations [11,12]. Acipimox can rapidly reduce the levels of free fatty acid (FFA), triglyceride, cholesterol, and low-density lipoprotein (LDL), and increase the level of high-density lipoprotein (HDL) in plasma [13,14,15]. Compared with nicotinic acid, acipimox has fewer side effects and is a clinically used first-line lipid-lowering drug, especially for the hyperlipidaemic patients that do not respond to other therapeutic regimens. Under the normoxic environment, acipimox is rapidly and almost completely absorbed from the gastrointestinal tract and hardly bound to plasma proteins [16]. Drug transporters also do not affect the absorption of acipimox. The drug is not significantly metabolized and elimination occurs by urinary excretion of the unchanged drug [17].

The previous study of our group found that the transcription level of the HCAR2 gene, the target of acipimox, in a plateau was 4.37 times higher than in a plain environment, but few researchers explored the changes in its pharmacokinetics and pharmacodynamics in the hypoxic environment. Based on the hypothesis, the expression changes of the target may affect drug pharmacokinetics, and this study attempted to explore the pharmacokinetics of acipimox in the hypoxic environment. In this paper, we first developed a novel, sensitive and specific method for the quantification of acipimox in rat plasma and tissues, and then successfully applied it to study the pharmacokinetics of acipimox in normoxic and hypoxic rats. The results provide important and valuable information for discovering and developing novel anti-hypoxia drugs, as well as a better understanding of the safety and efficacy of acipimox.

## 2. Results and Discussion

### 2.1. Method Development

Protein precipitation was tested for sample preparation in our test. Acetonitrile as precipitant could provide a satisfactory recovery (≥95%), and this method proved to be simple and suitable for the simultaneous determination of acipimox in rat plasma and tissues. Several attempts were made with different C_18_ HPLC columns, and a practical and common Shiseido Capcell PAK C_18_ column (100 mm × 2.1 mm, 5 μm) was applied to our study because the best chromatographic separation and response were achieved on this column. The concentration of aqueous 0.1% (*v*/*v*) ammonia solution proved to give a better response than other aqueous phases (e.g., for 0.1% formic acid). The gradient elution changed linearly from 85:15 (*v*/*v*) 0.1% ammonia aqueous solution-acetonitrile to 20:80 at a flow rate of 0.2 mL/min. A variety of compounds (such as nicotinic acid, carbamazepine, etc.) were tested to obtain satisfactory quantitative analysis results. Among them, acetylsalicylic acid was selected as IS because of its stability and high recovery.

**Figure 2:**
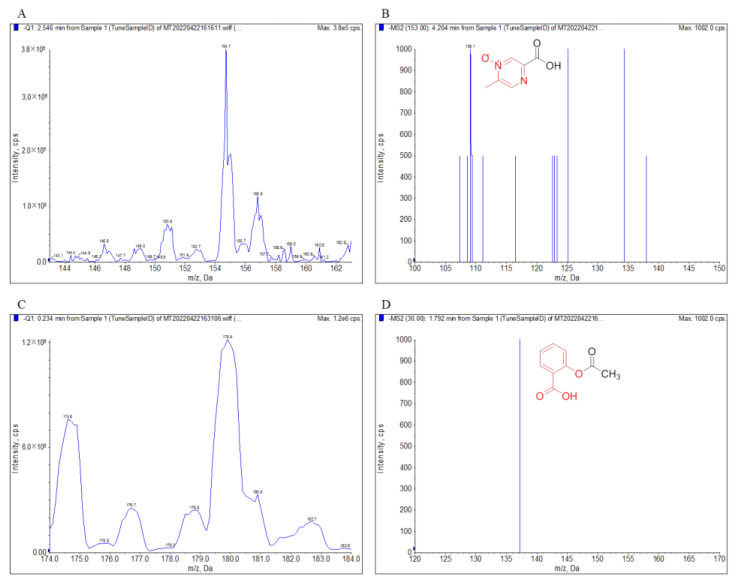
The MS Scan spectra, product ion spectra of [M−H]^−^ and their fragmentation pathways. The MS Scan spectra of acipimox (**A**), product ion spectra of [M−H]^−^ of acipimox (**B**), MS Scan spectra of IS (**C**), and product ion spectra of [M−H]^−^ of IS (**D**).

The MS2 scan mode was performed in positive or negative ion mode to optimize the ESI conditions of acipimox, IS, and found to achieve a better response in negative ionization mode. The capillary temperature, vaporizer temperature and flow rate were optimized to obtain protonated molecules of the analytes. The fragmentor and collision energy values were optimized, and the MRM transitions were monitored in order to obtain the maximum peak area. The mass spectrometry conditions were shown in Table 1. The precursor [M−H]^−^ of acipimox and IS were *m*/*z* 153.0 and 178.9, respectively. The ion *m*/*z* 109.1 and 137.3 were then selected as product ions of acipimox and IS in the product ion mode, respectively. Therefore, the MRM transitions were chosen to be *m*/*z* 153.0 → 109.1 for acipimox and *m*/*z* 178.9 → 137.3 for IS. The product ion mass spectra of [M−H]^−^ of acipimox and IS were presented in Figure 2.

### 2.2. Method Validation

#### 2.2.1. Selectivity

**Figure 3:**
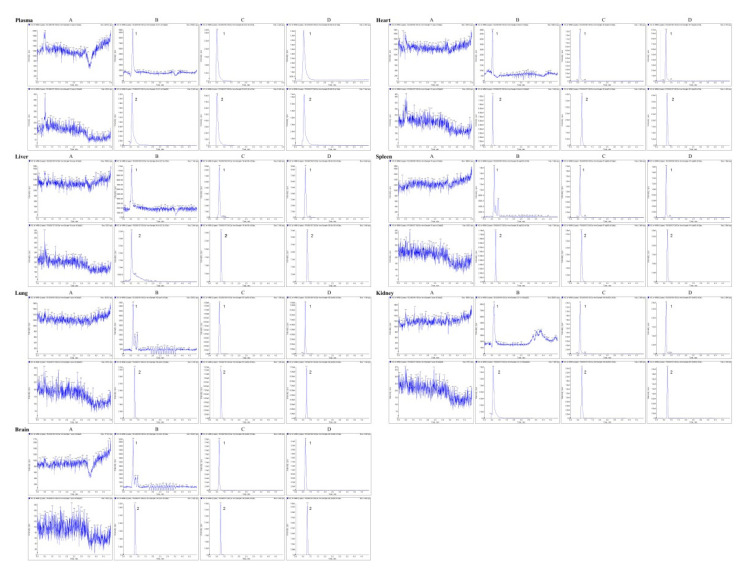
Typical multireaction monitoring chromatograms of acipimox and internal standard. (**A**) a blank plasma or tissue homogenate; (**B**) a blank plasma or tissue homogenate spiked with acipimox at LLOQ level (the concentrations shown in Table 2) and IS of 5 μg/mL; (**C**) a blank plasma or tissue homogenate spiked with acipimox (plasma: 25 μg/mL; heart, liver: 1 μg/mL; spleen, kidney, brain: 5 μg/mL; lung: 10 μg/mL) and IS (5 μg/mL); (**D**) a plasma or tissue homogenate sample collected 0.5 h after drug administration. 1: acipimox; 2: IS.

Representative chromatograms of blank plasma, plasma sample spiked with acipimox and IS, and a pharmacokinetic sample from SD rat 1 h after oral administration of acipimox are represented in Figure 3. Representative chromatograms of blank tissue homogenate, tissue homogenate sample spiked with acipimox and IS, and a pharmacokinetic sample from SD rat 1 h after oral administration of acipimox are also represented in Figure 3. The results showed that there were no endogenous substance peaks from the rat plasma and tissue homogenate samples and drug metabolite peaks interfering with the analytes and the IS at the retention times. The LC-MS/MS method described was thus selective and specific.

#### 2.2.2. Linearity and Lower Limit of Quantification

Quantification was based on the IS method of plotting the peak areas ratios of the analyte/IS versus the nominal plasma (or tissue homogenate) concentration of the test compound with 1/x^2^ as weighting factors, which was fitted by least square linear regression. Sensitivity was evaluated by determining the lower limit of quantification (LLOQ), which is defined as the lowest concentration that can be reliably and reproducibly measured in at least five replicates. The LLOQ had to have accuracy and precision of ≤20% and a signal/noise ratio ≥ 10. The equation, r^2^ values, linear range and LLOQ of the calibration curve in plasma and different tissue homogenates were shown in Table 2.

#### 2.2.3. Precision and Accuracy

The precision and accuracy of the method were assessed in rat plasma and tissue homogenate by performing replicate analyses of four QC samples (LLOQ, low, middle and high). The procedure was repeated in a single day (intra-day) and between three different days (inter-day) on the same spiked standard series. The data are shown in Table 3, indicating the accuracy and precision within the acceptable criteria for bioanalytical purposes (≤15%).

#### 2.2.4. Recovery and Matrix Effect

The recoveries were calculated at three concentrations by comparing the ratio of the responses of analytes spiked before the extracted process with that of extracted plasma and tissue homogenate samples in six replicates. The results are summarized in Table 4. The data indicated that the extraction recoveries at low, middle, high QC levels and IS were found to be reproducible, consistent and concentration independent in rat plasma and tissue homogenate.

Mean matrix effects of acipimox were in the range over 97.03–98.57% in plasma and 88.93–112.14% in the tissue homogenate, respectively. The recovery and matrix effect of IS were also investigated at 5 μg/mL. The results are shown in Table 4, indicating that no latent co-eluting endogenous substance interfered with the ionization of analytes and IS.

#### 2.2.5. Stability

A stability analysis of QC samples (low, medium and high) was performed by analyzing four different conditions. The data of the stability study showed that the variation in the concentration was within ±15% of the nominal concentration (Table 5), indicating that no significant degradation of any analyte occurred in rat plasma and tissue homogenate.

### 2.3. Pharmacokinetics Comparison

**Figure 4:**
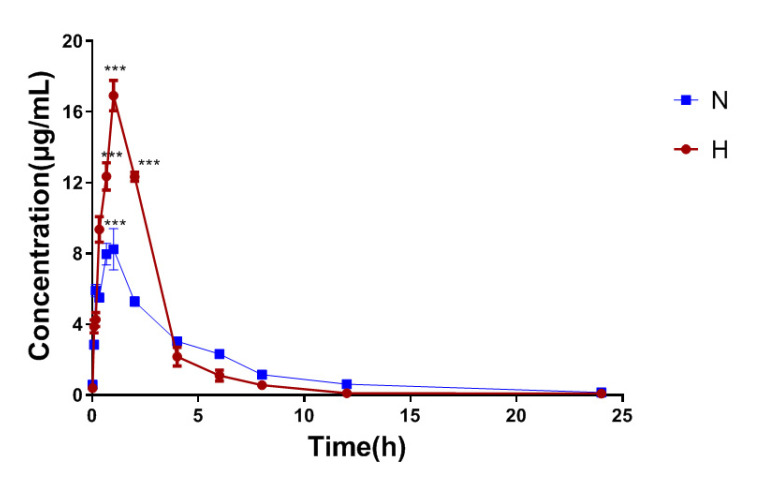
Mean plasma concentration-time curves of acipimox in rats with and without hypoxia following intragastric administration to rats (*n = 6*). N: Normoxia; H: Hypoxia; *** *p* < 0.001.

Under the high altitude hypoxia environment, a large amount of FFA accumulates in cells through the regulation of HIF, which further leads to lipid metabolism disorders. In clinical applications, acipimox regulates lipid metabolism disorders by reducing FFA as first-line therapy. However, to the best of our knowledge, there are no reports of the pharmacokinetic changes of acipimox at high altitude. Therefore, in this study, the method was applied to study the quantification of acipimox in rat plasma and tissue homogenate after full validation. Figure 4 shows the mean plasma concentration-time profiles of acipimox under normoxic and hypoxic conditions. The changes in the main pharmacokinetic parameters of acipimox influenced by hypoxia are shown in Table 6. From a comparison of the main pharmacokinetic parameters of acipimox between normoxic and hypoxic rats, the peak plasma concentration (Cmax), time to reach Cmax (Tmax) and area under the plasma drug concentration-time curve (AUC) of acipimox in hypoxic rats were markedly increased, and mean residence time (MRT), half-life (T_1/2_), clearance (CLz/F) and volume of distribution (Vz/F) were markedly decreased, which were statistically significant differences in these parameters (*p* < 0.05). The results showed that the content of acipimox in hypoxic rats was larger than in normoxic rats and the absorption and elimination were slower. The plasma concentration decreased rapidly in the initial stage under high altitude hypoxia, while the decline rate gradually slowed down in the later stage and the overall elimination time was prolonged. It might indicate that acipimox was rapidly distributed into tissues and organs under hypoxia, but slowly excreted out of the body. This result is consistent with the tissue distribution results, indicating that hypoxia significantly altered the main pharmacokinetics characteristics of acipimox in rats. Acipimox is not metabolized in vivo and is eliminated by its original form, but we found that its transcription level of the target gene (HCAR2) was markedly increased. Therefore, we speculate that the increased concentration of acipimox under hypoxic conditions may be due to the increased transcription level of HCAR2. Some studies have shown that hypoxia may affect blood circulation [5], vascular permeability [4] and gut flora [10], which may lead to the absorption and elimination changes of acipimox. Based on current in vivo experiments, the conclusions we have obtained still require further in vitro experiments for confirmation.

### 2.4. Tissue Distribution

**Figure 5:**
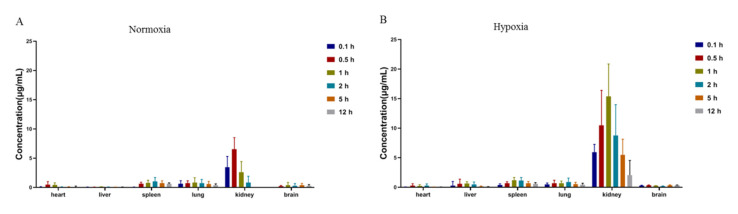
Tissue distribution of acipimox in rats with and without hypoxia after oral administration to rats (*n = 6*). (**A**) Tissue distribution of acipimox in normoxic rats; (**B**) Tissue distribution of acipimox in hypoxic rats.

The tissue distribution of acipimox in rats at 0.1, 0.5, 1, 2, 5 and 12 h after the oral administration is presented in Figure 5. At 0.1 h after administration, different concentrations of acipimox were detected in most of the rat tissues. The highest concentration level was observed in kidney, followed by spleen, lung, liver, heart and brain, indicating that acipimox could be distributed rapidly and widely in various tissues. The concentration of acipimox in kidney was significantly higher than in other tissues, which demonstrated that acipimox was mainly accumulated in the kidney, and renal excretion might be the main elimination route for acipimox. In addition, acipimox was detectable at a rather low concentration in the brain. However, the concentration did not change significantly in 2 h. This illustrated that acipimox could transfer across the blood-brain barrier, and be retained in the brain for a long time.

**Figure 6:**
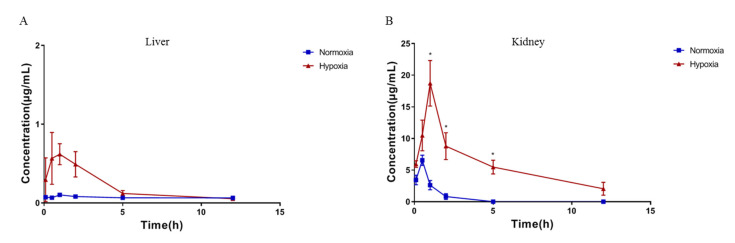
The concentration of acipimox in kidney and liver with and without hypoxia after oral administration to rats (*n = 6*). (**A**) Mean concentration-time curves of acipimox in liver; (**B**) Mean concentration-time curves of acipimox in kidney. * *p* < 0.05.

Compared to normoxia, the concentration of acipimox in kidney and liver were markedly increased in hypoxic rats (Figure 6). The reason may be that the concentration of acipimox in plasma increased under the hypoxic condition and that kidney is the main elimination route. We speculate that the increased concentration of acipimox in liver may be related to the up-regulation of HCAR2 in hypoxic rats. With the extent of the time, the concentrations of acipimox in most of the tissues decreased obviously after 0.5 h in normoxic rats, while it decreased obviously after 1 h in hypoxic rats. This indicates that the absorption of acipimox was slower under the hypoxic condition, which was congruent with the variation trend in the plasma concentration. Further experiments were required to verify whether the elevated concentration of acipimox in kidney and liver could cause toxicity in hypoxic rats.

### 2.5. Expression of Lipid Metabolism-Related Proteins and Changes of Plasma FFA

**Figure 7:**
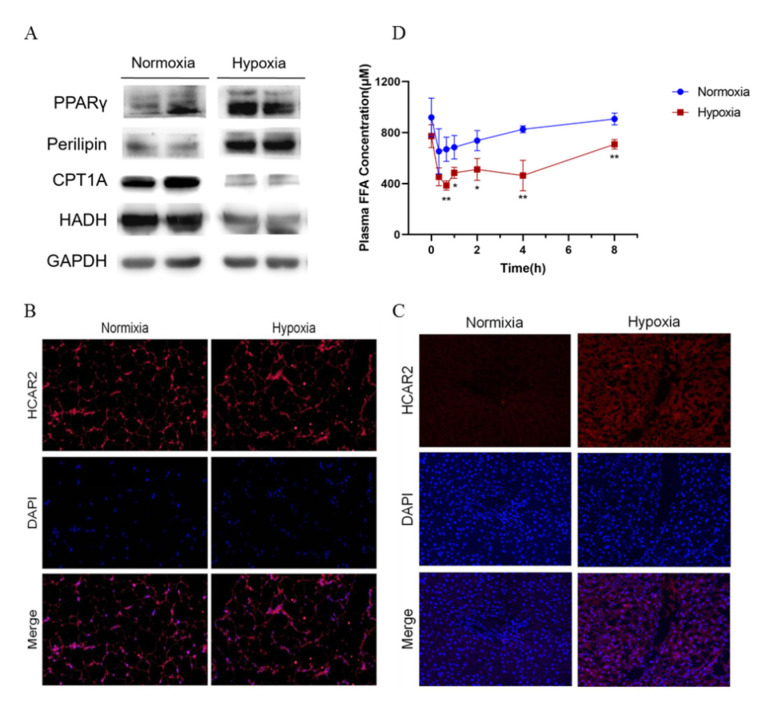
Effects of hypoxia on the expression of HCAR2 and lipid metabolism. (**A**) The expression changes of lipid metabolism-related proteins; (**B**) Immunofluorescence staining of HCAR2 in adipose tissue of normoxia or hypoxia rats; (**C**) Immunofluorescence staining of HCAR2 in liver of normoxia or hypoxia rats; (**D**) The changes of serum FFA in rats.

To investigate the effect of hypoxia on the metabolism of acipimox, the protein levels of key markers regulating lipid metabolism in liver were investigated. As shown in Figure 7A, compared to the normoxic group, the expressions of CPT1A and HADH in liver were down-regulation in hypoxic rats, while the expressions of PPARγ and perilipin were up-regulated. This indicates that the consumption of FFA by liver was reduced under the hypoxic environment and more FFA was stored in lipid droplets, which could lead to the accumulation of lipid droplets and decrease FFA in serum. Next, we used immunofluorescence staining to determine whether hypoxia increased the target protein expression of acipimox in liver and adipose tissue. HCAR2 expression in the normoxia group was lower than that in the hypoxia group (Figure 7B,C). Among them, the up-regulation was especially obvious in liver tissue. According to the metabolic characteristic of acipimox, we speculate that the metabolism changes of acipimox might be related to the expression changes of HCAR2 and the lipid metabolism-related proteins, the changes of blood flow to organs, and changes in gut microbiota. We will verify the hypothesis in future work.

To determine whether hypoxia affected the efficacy of acipimox, we investigated the levels of serum FFA in each group. The level of plasma FFA reduced significantly (*p* < 0.05) when acipimox was administered (Figure 7D). Furthermore, the levels of FFA decreased more in the hypoxia group than in the groups treated with normal oxygen concentration. Compared to normoxia rats, the concentration of acipimox in plasma was higher and the drug effect was stronger under the same dosage in hypoxia rats. It is indicated that the dosage of acipimox might be adjusted appropriately during clinical medication in hypoxia.

### 2.6. Toxicity

**Figure 8:**
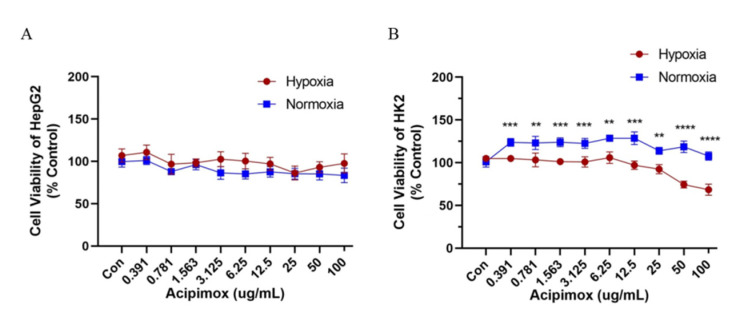
The cytotoxicity of acipimox on kidney and liver in normoxia and hypoxia condition. The effect of acipimox on proliferation in HepG2 (**A**) and HK-2 cells (**B**). ** *p* < 0.01, *** *p* < 0.001 and **** *p* < 0.0001.

To determine the cytotoxicity of acipimox on kidney and liver cells, HK-2 and HepG2 cells were exposed to various concentrations of acipimox (0.391–100 μg/mL, Figure 8) for 24 h. The CCK-8 result showed that acipimox did not affect the proliferation of HepG2 cells within 24 h both in normoxia and hypoxia conditions, while it inhibited the proliferation of HK-2 cells under hypoxia conditions. The effect of acipimox on HK-2 cells was dose-dependent. It indicated that the high concentration of acipimox had an effect on kidney cells and the dosage of acipimox might be reduced at high altitudes.

## 3. Materials and Methods

### 3.1. Chemicals and Reagents

Reference standards of acipimox and acetylsalicylic acid (IS) with purity (>99.00%) were purchased from the National Institute for the Control of Pharmaceuticals and Biological Products (Beijing, China). HPLC-grade acetonitrile was obtained from Thermo Fisher Scientific (Waltham, MA, USA). The ammonia of HPLC grade was obtained from Sigma-Aldrich (St. Louis, MO, USA). The water used in the experiment was double distilled. All other chemicals were of the highest commercially available grade.

### 3.2. Animals

Male Sprague–Dawley rats aged seven to eight weeks, weighing 250 ± 20 g, were purchased from the Charles River (Beijing, China). All rats were housed internally in flawless animal rooms maintained at constant temperature (23 °C ± 2 °C) and humidity (55% ± 10%), and a 12 h light/dark cycle. The rats had unrestricted access to food and water. Animal studies were carried out in accordance with the Guide for the Care and Use of Laboratory Animals as adopted and promulgated by the National Health Ministry of China.

Protocols of animal experiments had been approved by Animal Center of Beijing Institute of Radiation Medicine. Twelve rats were randomly divided into two groups: (1) Normoxia group (*n = 6*): exposed to the normal air condition (21% O_2_) and received 80 mg/Kg acipimox; (2) Hypoxia group (*n = 6*): simulated the hypoxic air condition of 4300 m altitude (13% O_2_) and received 80 mg/Kg acipimox after seven d. The blood samples were collected in tubes containing sodium heparin at 0, 0.0167, 0.0833, 0.167, 0.333, 0.667, 1, 2, 4, 6, 8, 12 and 24 h after dosing; the tissue samples were collected at 0.1, 0.5, 1, 2, 5 and 12 h after dosing.

### 3.3. LC-MS/MS Conditions

Quantitative analysis was achieved using a Shiseido liquid chromatograph (NANOSPACE SI-2 3301) consisting of a quaternary pump, temperature controlled column compartment and flow-through needle autosampler. Chromatographic separation was conducted using a Shiseido Capcell PAK C18 MG III column (100 mm × 2.1 mm, 5 μm) at 30 °C through a column oven. The mobile phase solutions were composed of water containing 0.1% (*v*/*v*) ammonia (A) and acetonitrile (B). Initial gradient conditions were 85:15 (A:B). From the 2 to 2.5 min the B was increased to 80% and reverted the initial gradient conditions from 3.5 min. The total run time was 5 min. The mobile phase was returned to the initial conditions and reequilibration for a period of time. The flow rate was 0.2 mL/min. The sample injection volume was 2 μL.

The AB-4000 triple quadrupole mass spectrometer (Applied Biosystem, Waltham, MA, USA) was combined with an electrospray ionization (ESI) source. The parameters of the mass spectrometer are as follows: capillary voltage: 4.5 kV, drying gas temperature: 350 °C, drying gas flow: 12 L/min, nebulizer pressure: 45 psi, corona current:10 nA, sheath gas flow:10 L/min and sheath gas temperature: 350 °C. Multireaction monitoring (MRM) analysis using a negative ion ESI mode was used to monitor ion transitions of *m/z* 153.0 → 109.1 for acipimox, 178.9 → 137.3 for acetylsalicylic acid. The fragmentor (Frag) values set for acipimox and IS were 120 and 100 V, respectively. The collision energy values set for acipimox and IS were −13.5 and −9 eV, respectively. The software Analyst was used to acquire and process data (Analyst 1.6.3, Applied Biosystem, Waltham, MA, USA).

### 3.4. Stock Solutions, Calibration Standards and Quality Control Samples

The stock standard solutions of acipimox (1 mg/mL) and IS (1 mg/mL) were prepared by acetonitrile and then stored in the dark at −20 °C. The stock solution of acipimox was diluted with acetonitrile to prepare the series of working solutions for the analytes, and the IS stock solution was also diluted to the concentration of 50 μg/mL with acetonitrile. Working solutions for calibration standards were prepared by spiking the appropriate amount of the standard working solution into the blank rat plasma to give the nominal concentration range of 0.1–50 μg/mL for acipimox. The linearity of acipimox in different tissue homogenates (heart, liver: 0.05–5 μg/mL; spleen, lung: 0.1–25 μg/mL; kidney: 0.1–50 μg/mL; brain: 0.05–10 μg/mL) were also assessed using a seven-point calibration curve of the diluted standards. Three levels of QC samples in plasma and different tissue homogenates were prepared in the same way as the calibration standards.

### 3.5. Samples Preparation

100 μL of plasma sample (or tissue homogenate), spiked with 10 μL of IS solution (5 μg/mL), was spun for 30 s at room temperature. Next, 1 mL of acetonitrile was added into the mixture. After vortex mixing for 30 s, the mixed sample was centrifuged at 12,000 rpm for 15 min at 4 °C. 800 μL of the supernatant was transferred to another centrifuge tube and dried with nitrogen at 30 °C. The residue was redissolved with 100 μL acetonitrile and centrifuged at 12,000 rpm for 15 min at 4 °C after being vortexed for 3 min. The supernatant was then transferred into an autosampler vial, and 2 μL aliquot was injected into the LC-MS/MS system for analysis.

### 3.6. Method Validation

The method validation procedure was conducted according to the guiding principles of the US FDA and EMA.

#### 3.6.1. Selectivity and Specificity

The selectivity of the method was evaluated with the retention time of analytes and IS by comparing chromatograms of blank plasma and plasma-spiked analytes for excluding endogenous material interference. To evaluate the specificity, the samples of blank plasma were analyzed by comparing with pharmacokinetic samples and the plasma-spiked analytes. The average response of endogenous interference is less than 20% of the analytes, and then the method was considered to be specific.

#### 3.6.2. Linearity and Lower Limit of Quantification

A calibration curve was set up by calculating the peak area ratios (y) of the analyte to IS versus the concentrations of the analytes (x) using a 1/x^2^ weighted linear least squares regression. The concentration of each analyte was determined by using the equations of linear regression obtained from the calibration curves. LLOQ was determined as the lowest concentration of the calibration curve with the signal/noise ratio being not less than 10. The accuracy and precision of each LLOQ sample should be less than 20%.

#### 3.6.3. Precision and Accuracy

Accuracy and precision were assessed in six replicates of four QC samples (LLOQ, low, middle and high) in a single day (intra-day) and four batches in three days in a row (inter-day), respectively. Acceptable limits for intra- and inter-day precision and accuracy were set at ±15%.

#### 3.6.4. Recovery and Matrix Effect

Recoveries of acipimox were determined by comparing the peak areas of the extracted QC samples with the peak areas of post-extracted blank plasma spiked at the corresponding concentrations. The matrix effects were assessed by the ratio of the peak area of extracted blank plasma spiked post extraction with analytes to those for the clean standard solutions at three concentration levels. The recovery and matrix effect of IS were also investigated at 5 μg/mL.

#### 3.6.5. Stability

A stability analysis of QC samples (low, medium and high) was performed by analyzing four conditions under different conditions: at room temperature for 12 h after preparation, extracted samples were kept at 4 °C for 24 h, samples after three freeze–thaw cycles, and samples stored at −80 °C for 30 days. An acceptable stability was defined as ≤15% loss of the initial drug concentration.

### 3.7. Pharmacokinetic and Tissue Distribution Comparison

All samples were subsequently treated as in the biological sample preparation procedure (3.5) and assayed by the LC–MS/MS method. The pharmacokinetic analysis was performed using the noncompartmental model analysis. The pharmacokinetic analysis was evaluated through Drug and Statistics version 2.2 (DAS 2.2, Shaanxi Energy Institute, Xianyang, China). The tissue distribution comparison used One-way ANOVA for statistical comparison between different groups with Prism software (GraphPad Software Inc. Version 8, San Diego, CA, USA).

The time to peak plasma concentration (T_max_) and the maximum plasma concentration (C_max_) could be obtained directly by observation. The total area under the plasma concentration-time curve (AUC) was calculated by the linear trapezoidal rule. The half-life (t_1/2_) is calculated as follows: t_1/2_ = ln2/ke. The clearance (CL) of the drug was obtained by the following formula: CL = Dose_(oral)_/AUC.

### 3.8. Western Blot Analysis

After treatment, rats in each group were used for the preparation of protein extracts. Total proteins were extracted from liver or adipose tissues samples using the radioimmunoprecipitation assay (RIPA) buffer (Beyotime, Shanghai, China) supplemented with phenylmethanesulfonyl fluoride (1 mmol/L; Sigma) and a protease and phosphatase inhibitor cocktail (100×; Thermo Fisher Scientific, Waltham, MA, USA). Equal amounts of protein samples were separated using 10% (*v*/*v*) sodium dodecyl sulfate-polyacrylamide gel electrophoresis (SDS-PAGE) and transferred onto a polyvinylidenedifluoride (PVDF) membrane. After washing, the membranes were incubated overnight at 4 °C with one of the following primary antibodies: rabbit polyclonal antibodies against CPT1A (1:1000; Abcam, Cambridge, UK), HADH (1:1000; Proteintech, Rosemont, IL, USA), PPARγ, Perilipin, GAPDH (1:1000; Cell Signaling Technology, Danvers, MA, USA). After further washing, the membranes were incubated for 1 h with corresponding horseradish peroxidase conjugated secondary antibodies (anti-rabbit IgG or anti-mouse IgG, 1:10,000; Abcam, UK). The immunoreactive bands were visualized using an enhanced chemiluminescent substrate (MILLIPORE, Burlington, MA, USA) with a GE ImageQuant LAS 500 (GE Healthcare, USA). The intensity of the protein bands was quantitated using a Gel Doc XR system (Bio-Rad, Hercules, CA, USA).

### 3.9. Confocal Laser Scanning Fluorescence Microscopy

At the end of treatment, a part of the liver and adipose tissue was fixed with 4% paraformaldehyde, embedded in paraffin, and sequentially sectioned. The tissues were permeabilized with 1% (*v*/*v*) TritonX-100 in PBS for 10 min at room temperature, and then blocked with goat serum for 1 h. After washing, the tissues were incubated overnight with anti-HCAR2 antibody (1:100) in goat serum and then incubated for 1 h with fluorescein isothiocyanate conjugated secondary antibody (Cy3 Conjugated, 1:100, Abcam, UK). The coverslips were mounted on glass slides with antifade mounting media (Invitrogen, Carlsbad, CA, USA), and the images were collected using an Olympus confocal microscope model FV1000 at 800 × 600 pixel resolution with a 60× objective lens (Carl Zeiss, Oberkochen, Germany). No fluorescence crossover was observed between the channels, and images were collected separately using the appropriate laser excitation wavelength and then merged.

### 3.10. Serum FFA Analyses

After seven days of hypoxia, blood samples in each group were collected to separate the serum and blood after received 80 mg/Kg acipimox. Fasting serum levels of free fatty acids (FFAs)in each rat were assessed using the corresponding commercial kits (MEIMIAN, Guangzhou, China). After processing the samples according to the instructions, we measured the absorbance at 450 nm using a microplate spectrophotometer (PerkinElmer, Waltham, MA, USA), and calculated the FFA content.

### 3.11. Cell Culture

The HepG2 and HK2 cells were purchased from the cell bank of the Chinese Academy of Sciences (Shanghai, China). The cells were maintained at 37 °C in the presence of 95% O_2_ and 5% CO_2_ in basic Dulbecco’s Modified Eagle’s medium (DMEM) supplemented with 10% fetal bovine serum (FBS) and antibiotics. The culture media was routinely changed after every two days, and the cells were passaged by trypsinization before reaching confluence.

The cells were also divided into two groups for different treatments: (1) Normoxia group (*n = 6*): exposed to the normal culture condition (95% O_2_ and 5% CO_2_)and treated with acipimox; (2) Hypoxia group (*n = 6*): simulated the hypoxic condition (1% O_2_ and 5% CO_2_) and treated with acipimox after 24 h.

### 3.12. Cell Viability Assay

Cell viability was detected using a Cell Counting kit-8 assay (CCK-8, Dojindo, Japan) according to the manufacturer’s instructions. 6000 cells were seeded in a 96-well plate per well. After growing for 24 h, cells were treated with gradient concentrations of acipimox (0–100 μg/mL) and treated with different oxygen concentrations. After 24 h, cells were washed with PBS buffer gently and incubated with DMEM medium containing 10% CCK-8 solution for 2 h in an incubator at 37 °C. The absorbance of each well was then determined with a microplate reader at 450 nm (Multiskan MK3, Thermo Fisher Scientific, Waltham, MA, USA). The cell viability of the treatment group was expressed as a percentage relative to that of the control group.

### 3.13. Statistical Analysis

All results were presented as mean ± standard deviation (SD) and generated from at least three independent experiments. Comparisons between the groups of rats were performed using an independent-samples Tukey’s test. *p* < 0.05 was considered statistically significant.

## 4. Conclusions

We have established and validated a novel LC-MS/MS method for the determination of acipimox in rat plasma and tissue homogenate, and successfully applied to characterize and compare the pharmacokinetics of acipimox in normoxic and hypoxic rats. The results showed that hypoxia significantly altered the in vivo pharmacokinetic characteristics of acipimox after oral administration of the drug in rats. The elevated expression of the acipimox target may be responsible for these changes under hypoxia conditions, which was also validated by the pharmacology and molecular biology results. The changes of pharmacokinetic parameters were in favor of treatment of some effects related to hypoxia. These results provide important and valuable information for guidance on high altitude medication, and combined with the results of a cell viability assay, provide a better understanding of the safety and efficacy of acipimox in hypoxia clinical practice.

## Figures and Tables

**Table 1:** The mass spectrometry conditions of acipimox and IS.

Mass Spectrometry Conditions	Acipimox	Acetylsalicylic Acid (IS)
Declustering Potential	−45 v	−25 v
Entrance Potential	−10 v	−16 v
Collision Energy	−13.5 v	−9 v
Collision Cell Exit Potential	−8 v	−8 v

**Table 2:** Standard curves of acipimox in plasma and tissue of rats.

Sample	Regression Equations	Linear Range (μg/mL)	r^2^	LLOQ (μg/mL)
Plasma	y = 0.079 x + 0.0276	0.1~50	0.9924	0.1
Heart	y = 0.0103 x + 0.00546	0.05~5	0.9900	0.05
Liver	y = 0.436 x − 0.0187	0.05~5	0.9915	0.05
Spleen	y = 0.0117 x + 0.000168	0.1~25	0.9940	0.1
Lung	y = 0.00943 x + 0.00325	0.1~25	0.9902	0.1
Kidney	y = 0.0137 x + 0.0287	0.1~50	0.9919	0.1
Brain	y = 0.012 x + 0.0039	0.05~10	0.9917	0.05

**Table 3:** Precision and accuracy for the analysis of acipimox.

Analyte	Spiked Conc. (μg/mL)	Intra-Day (*n = 6*)			Inter-Day (*n = 3 × 6*)		
		Measured Conc. (mean ± SD)	Precision (%RSD)	Accuracy (%RE)	Measured Conc. (mean ± SD)	Precision (%RSD)	Accuracy (%RE)
Plasma	0.1	0.10 ± 0.01	14.22	−4.70	0.09 ± 0.01	13.91	−8.45
	0.3	0.35 ± 0.01	3.52	14.98	0.34 ± 0.03	8.58	14.78
	20	20.85 ± 0.85	4.08	4.26	20.38 ± 1.07	5.26	1.90
	40	34.97 ± 3.00	8.59	−12.57	35.82 ± 2.24	6.26	−10.45
Heart	0.05	0.06 ± 0.01	18.78	13.84	0.05 ± 0.01	11.88	3.28
	0.15	0.16 ± 0.02	13.67	5.85	0.16 ± 0.01	3.28	4.26
	2	2.02 ± 0.17	8.45	1.15	2.09 ± 0.16	7.52	4.67
	4	4.07 ± 0.51	12.49	1.78	3.96 ± 0.35	8.89	−0.89
Liver	0.05	0.05 ± 0.00	2.91	8.88	0.05 ± 0.01	16.48	8.10
	0.15	0.14 ± 0.01	5.03	−5.14	0.16 ± 0.02	14.91	5.69
	2	2.01 ± 0.20	9.87	0.25	2.08 ± 0.20	9.52	3.88
	4	4.37 ± 0.48	11.05	9.25	3.78 ± 0.33	8.82	−5.51
Spleen	0.1	0.11 ± 0.01	11.05	6.33	0.10 ± 0.01	14.69	−1.09
	0.3	0.32 ± 0.01	4.04	8.30	0.33 ± 0.05	13.93	8.96
	12	11.60 ± 0.10	0.86	−3.31	12.04 ± 1.09	9.03	0.30
	20	18.53 ± 2.39	12.88	−7.37	21.95 ± 2.59	11.81	9.74
Lung	0.1	0.10 ± 0.00	1.19	3.59	0.11 ± 0.01	12.29	9.37
	0.3	0.30 ± 0.04	11.84	1.58	0.32 ± 0.04	13.80	5.61
	12	13.08 ± 1.53	11.70	9.00	12.48 ± 1.18	9.43	4.01
	20	21.00 ± 1.39	6.64	4.98	19.71 ± 1.86	9.42	−1.45
Kidney	0.1	0.10 ± 0.01	5.33	4.48	0.11 ± 0.02	14.99	6.72
	0.3	0.34 ± 0.02	6.92	12.46	0.33 ± 0.04	11.94	11.22
	20	21.81 ± 2.06	9.46	9.05	22.42 ± 1.49	6.63	12.12
	40	42.60 ± 5.79	13.59	6.51	42.44 ± 4.60	10.85	6.11
Brain	0.05	0.06 ± 0.00	2.03	16.75	0.05 ± 0.01	14.51	7.49
	0.15	0.17 ± 0.02	13.46	12.79	0.16 ± 0.02	9.90	9.49
	4	4.08 ± 0.22	5.37	1.92	4.03 ± 0.36	8.97	0.75
	8	8.51 ± 0.67	7.83	6.40	8.61 ± 0.46	5.29	7.60

RSD: relative standard deviation; RE: Relative error.

**Table 4:** Recovery and matrix effect of acipimox in plasma and tissue of rats (*n = 6*).

Analyte	Spiked Conc. (μg/mL)	Recovery		Matrix Effect	
		(Mean ± SD%)	RSD (%)	(Mean ± SD%)	RSD (%)
Plasma	0.3	101.53 ± 8.68	8.55	98.25 ± 8.42	8.57
	20	100.38 ± 4.99	4.97	97.03 ± 5.04	5.19
	40	101.90 ± 6.16	6.04	98.57 ± 2.64	2.68
	5 (IS)	101.49 ± 0.71	0.70	100.62 ± 2.33	2.31
Heart	0.15	95.87 ± 6.73	7.02	94.68 ± 7.43	7.85
	2	98.59 ± 5.57	5.65	98.16 ± 4.73	4.82
	4	89.28 ± 4.31	4.82	93.61 ± 6.48	6.92
	5 (IS)	105.90 ± 7.67	7.24	98.57 ± 6.12	6.20
Liver	0.15	106.96 ± 12.78	11.95	95.74 ± 3.36	3.51
	2	106.58 ± 4.51	4.23	112.14 ± 8.17	7.29
	4	97.36 ± 2.77	2.85	98.63 ± 11.29	11.45
	5 (IS)	101.36 ± 6.32	6.23	93.46 ± 6.60	7.06
Spleen	0.3	105.08 ± 6.42	6.11	98.15 ± 4.87	4.96
	12	104.96 ± 4.95	4.71	94.87 ± 9.41	9.92
	20	90.29 ± 10.42	11.54	95.74 ± 8.14	8.50
	5 (IS)	104.79 ± 7.20	6.87	99.81 ± 6.60	6.61
Lung	0.3	100.58 ± 6.12	6.09	93.86 ± 5.29	5.64
	12	91.60 ± 9.66	10.54	101.00 ± 2.37	2.34
	20	93.47 ± 7.83	8.38	88.93 ± 8.80	9.90
	5 (IS)	94.35 ± 8.50	9.01	93.47 ± 2.93	3.14
Kidney	0.3	94.30 ± 5.32	5.64	101.33 ± 9.77	9.64
	20	95.78 ± 12.71	13.27	98.05 ± 6.24	6.36
	40	109.34 ± 7.96	7.28	94.22 ± 1.70	1.80
	5 (IS)	98.67 ± 5.98	6.06	99.43 ± 7.20	7.24
Brain	0.15	91.39 ± 2.78	3.04	96.90 ± 13.09	13.51
	4	100.86 ± 7.88	7.81	95.76 ± 1.60	1.67
	8	101.75 ± 7.38	7.25	96.07 ± 2.17	2.26
	5 (IS)	97.75 ± 4.90	5.02	95.64 ± 8.02	8.39

IS: Internal standard.

**Table 5:** Stability of Acipimox in the plasma and tissue of rats (*n = 6*).

Analyte	Spiked Conc. (μg/mL)	Postpreparation 12 h at Room Temperature		Auto-Sampler Stability at 4 °C for 24 h		Frozen (−80 °C) for 30 Days		Three Freeze–Thaw Cycles	
		Measured Conc. (μg/mL)	Accuracy (%)	Measured Conc. (μg/mL)	Accuracy (%)	Measured Conc. (μg/mL)	Accuracy (%)	Measured Conc. (μg/mL)	Accuracy (%)
Plasma	0.3	0.34 ± 0.05	14.59	0.34 ± 0.01	14.96	0.30 ± 0.06	−0.48	0.28 ± 0.06	−7.48
	20	20.46 ± 0.94	2.28	20.30 ± 1.40	−1.52	20.10 ± 0.14	0.46	20.20 ± 0.57	1.02
	40	36.43 ± 1.19	−8.92	35.21 ± 3.17	−11.98	45.49 ± 5.47	13.72	40.39 ± 7.93	0.97
Heart	0.15	0.16 ± 0.00	6.56	0.15 ± 0.01	−2.67	0.14 ± 0.07	−7.36	0.13 ± 0.00	−10.63
	2	2.22 ± 0.04	11.10	1.89 ± 0.37	−5.72	2.10 ± 0.17	4.75	1.86 ± 0.49	−7.22
	4	3.92 ± 0.47	−1.92	4.48 ± 0.36	12.09	3.54 ± 2.94	−11.42	4.15 ± 0.27	3.85
Liver	0.15	0.15 ± 0.01	3.31	0.15 ± 0.02	2.43	0.13 ± 0.02	−11.85	0.15 ± 0.02	−1.96
	2	2.27 ± 0.40	13.59	2.21 ± 0.61	10.27	1.92 ± 0.24	−3.95	2.14 ± 0.27	6.93
	4	3.87 ± 0.34	−3.30	4.08 ± 0.41	2.06	3.82 ± 0.31	−4.58	3.84 ± 0.46	−3.95
Spleen	0.3	0.33 ± 0.04	10.44	0.33 ± 0.03	9.27	0.28 ± 0.03	−5.95	0.31 ± 0.04	2.27
	12	11.52 ± 1.27	−4.00	11.61 ± 0.14	−3.25	11.44 ± 1.31	−4.69	11.77 ± 1.27	−1.88
	20	18.13 ± 1.90	−9.33	20.99 ± 1.02	4.95	20.10 ± 3.00	0.50	17.74 ± 2.65	−11.28
Lung	0.3	0.31 ± 0.01	4.89	0.32 ± 0.03	8.33	0.34 ± 0.09	12.36	0.29 ± 0.02	−3.74
	12	12.05 ± 0.65	0.43	10.44 ± 5.51	−12.99	11.62 ± 1.70	−3.13	11.24 ± 2.91	−6.33
	20	19.33 ± 2.53	−3.36	20.94 ± 0.50	4.72	18.15 ± 3.73	−9.22	18.95 ± 2.02	−5.25
	0.3	0.32 ± 0.04	5.68	0.33 ± 0.03	9.78	0.33 ± 0.06	8.85	0.32 ± 0.02	8.25
Kidney	20	22.52 ± 3.05	12.60	22.56 ± 4.57	12.78	18.25 ± 2.28	−8.76	18.99 ± 0.70	−5.07
	40	41.88 ± 5.57	4.69	41.53 ± 1.05	3.83	39.60 ± 2.70	−1.00	41.68 ± 2.34	4.21
	0.15	0.14 ± 0.02	−5.10	0.14 ± 0.01	−3.97	0.15 ± 0.02	2.91	0.15 ± 0.02	−2.27
Brain	4	3.90 ± 0.34	−2.51	4.24 ± 0.58	6.10	3.62 ± 0.15	−9.43	3.92 ± 0.46	−2.10
	8	8.56 ± 0.74	7.06	8.69 ± 0.18	8.59	8.03 ± 0.60	0.36	7.66 ± 1.24	−4.24
	0.3	0.34 ± 0.05	14.59	0.34 ± 0.01	14.96	0.30 ± 0.06	−0.48	0.28 ± 0.06	−7.48

**Table 6:** Pharmacokinetic parameters of acipimox to rats with and without hypoxia (*n = 6*).

Parameter	Normoxia	Hypoxia
AUC_(0−t)_ (μg/mL × h)	38.338 ± 2.34	46.587 ± 6.085 ***
MRT_(0−t)_ (h)	4.774 ± 0.45	2.46 ± 0.43 ***
C_max_ (μg/mL)	9.071 ± 2.39	16.923 ± 2.43 ***
t_1/2_ (h)	4.394 ± 0.608	1.877 ± 1.121 ***
T_max_ (h)	0.584 ± 0.445	0.917 ± 0.154 *
CLz/F (L/h/kg)	0.512 ± 0.029	0.375 ± 0.16 *
Vz/F (L/kg)	3.254 ± 0.533	0.972 ± 0.727 ***

Data are given as the mean ± SD; * *p* < 0.05, and *** *p* < 0.001 proved to be significantly different between the normoxic and hypoxic groups.

## Data Availability

The data presented in this study are available on request from the corresponding author. The data are not publicly available due to privacy restriction.

## Sample Availability

Samples of the compounds acipimox are available from the authors.
